# Inpatient Treatment of Community-Acquired Pneumonias with Integrative Medicine

**DOI:** 10.1155/2013/578274

**Published:** 2013-05-22

**Authors:** Ulrich Geyer, Klas Diederich, Maria Kusserow, Andreas Laubersheimer, Klaus Kramer

**Affiliations:** ^1^Department of Homeotherapy, Heidenheim Hospital, Teaching Hospital of The University of Ulm, Schloßhaustraße 100, 89522 Heidenheim, Germany; ^2^Departement of Mathematics, University of Wuppertal, 42119 Wuppertal, Germany; ^3^Departement of CI, University of Ulm, 89081 Ulm, Germany

## Abstract

*Introduction.* The aim of the presented observational case series was to evaluate the experience in treating patients with community-acquired pneumonia (CAP) within integrative medicine, particularly anthroposophic medicine in a well-experienced and specialized unit. *Patients and Methods.* Patients with proven CAP were evaluated (CAP-study group) based on a retrospective chart review. To estimate the severity of pneumonia, the pneumonia severity index (PSI) was applied. Treatment efficacy was evaluated regarding body temperature, CRP level, leukocytes blood count, the need to be treated on ICU, and mortality. Results were compared with the inpatient data of the Pneumonia PORT Validation Cohort. *Results.* 15/18 patients of the CAP-study group belonged to risk class groups I–III (low and moderate risk), 2 patients to risk class IV, and one patient to risk class V (severe pneumonia). 16/18 patients were treated with anthroposophic medicine only and 2/18 got additionally antibiotic therapy (both of risk class IV). A significant reduction of body temperature, CRP level, and leukocytes blood count has been obtained by applying anthroposophic medicine, while neither complications nor pneumonia-related death occurred. Compared with the control group there was no significant difference in mortality rate, whereby no patient had to be treated on the ICU, but the duration of hospital stay was significantly longer in the presented series. *Conclusion.* Inpatient treatment of CAP with anthroposophic medicine without the use of antibiotics may achieve reasonable results in selected cases. Additional larger sized prospective controlled trials should further clarify the role of AM in the treatment of CAP.

## 1. Introduction

Optimal treatment of pneumonia plays a critical role in temporary medicine regarding morbidity and mortality [1–4]. In Germany, annual occurrence of pneumonias accounts for 400000 to 600000 patients, with an inpatient treatment rate of 30–50% [5]. Lethality amounts to 0.6% among outpatients and from 13 to 14% among inpatients whereby a significant age dependency is typical [4, 6].

According to treatment guidelines, applications of antibiotics or other specific agents are strongly recommended. The aim of these standard treatments is to eliminate the causative agent (bacteria, viruses or mycoides, etc.) [4]. With increasing resistance to antibiotics [7–11], alternative treatment options are under debate. Moreover, the increasing request of patients on alternative treatment options [12–22] as well as cumulating data which might indicate a potential anticancerous role of acute inflammatory diseases and/or an adverse effect in antibiotic treatment [23–31] is triggering the discussion regarding treatment efficacy. In contrast, some approaches of integrative medicine primarily intend to support the human resources of recovery for curation (*“aspect of salutogeneses”*), while reducing or eliminating the causative agents (bacteria, viruses, or mycoids) becomes a secondary result only.

However, data on treatment efficacy in pneumonias including complementary and alternative medicine (CAM)—in particular anthroposophic medicine (AM)—are limited.

The aim of the presented study is to evaluate the treatment experience in applying anthroposophic medicine on a specialized and experienced unit with focus on the treatment of pneumonia. 

## 2. Patients and Methods

Patients with proven diagnosis of community-acquired pneumonia (CAP), according to current guidelines [4], who were treated within the Department of Homeotherapy in Heidenheim between March 1999 and September 2001 were registered and consecutively divided into five subgroups. There were no further selection criteria, despite the willingness and consent of the patients, who were requesting integrative treatment. The Department of Homeotherapy in the Hospital of Heidenheim (Teaching Hospital of the University of Ulm, Germany) looks back on a 65-year experience in practising anthroposophic medicine (AM) including a broad spectrum of different applications within the scope of integrative medicine (IM). The concept of integrative medicine seeks not to weigh up conventional and alternative medicine against each other but to optimize both forms of treatment while intending an individualized approach [14–16, 21].

Chart review was carried out focusing on the following parameters: initial clinical symptoms, radiologic features, blood sample tests, and clinical followup. Clinical data were retrospectively reviewed based on the hospital records including medical history and on results from the contributing radiologists and laboratory.

According to current guidelines [4] the diagnostic criteria for CAP were the clinical picture of an acute pneumonia, such as possible fever, shivering, cough, phlegm, sputum, chest pain, dyspnea in association with increased leukocyte and/or CRP levels, and newly manifest infiltration in a chest X-ray [4]. Patients with atypical manifestations, particularly elderly people, were also included if a clinical change occurred, like confusion or mobility impairment which could not be explained by any other reason, but at the same time a newly manifest infiltrate had to be spotted on the chest X-ray [4]. All patients who did not fulfill these criteria, who had hospital-acquired pneumonia (HAP), or who had immune deficiency were excluded. Also, lost of followup was a reason for noninclusion. 

Results of chest X-rays were reviewed by two—and for this case series reevaluated by additional one—independent consultant radiologist(s) who were blinded concerning prior diagnosis but confirming radiological signs of pneumonia.

**Figure 1:**
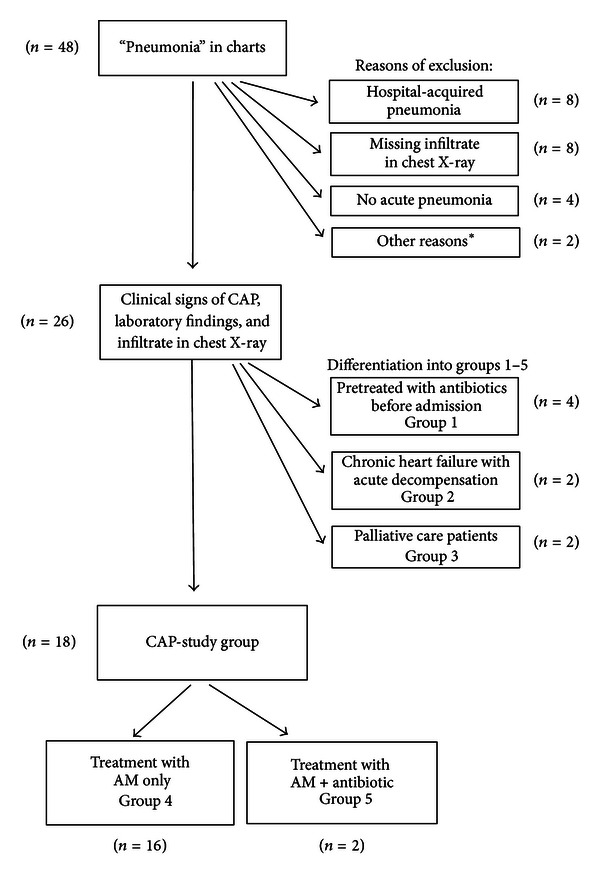
Flow chart of the inclusion and exclusion processes. *Other reasons for exclusion: patients with an immunodeficiency (*n* = 1), patients lost of followup (*n* = 1, this patient wanted to be moved to a hospital closer to home).

In order to reduce potential coaffecting circumstances five different groups were differentiated (Figure 1).

Group 1 includes patients pretreated with antibiotics before admission to the Department of Homeotherapy; group 2 includes patients with an acute cardiac decompensation and a congestive pneumonia (treatment of heart failure improves usually pneumonia too in these cases); group 3 includes patients in palliative care. All other patients were defined as the *CAP-study group*: treated either with AM only (group 4) or additionally with antibiotics (group 5).

Pneumonia severity index (PSI) was applied in order to indicate the severity level of pneumonia, divided into five risk classes [32–35] (Table 1).

As shown in Table 1, patients are scored between −10 and +30 points for the different parameters. Patients were assigned to a risk class (risk class II, III, IV, or V) according to the number of points they scored. Identifying patients in risk class I is extensively described by Fine et al. [32]. Fine et al. had derived a prediction rule for the prognosis by analysing data of 14, 199 adult inpatients with CAP. This risk score was validated on 38,039 adults hospitalized and data of 2287 inpatients and outpatients with community-acquired pneumonia.

In case of missing classification data, only the available information were incorporated into risk assessments. Consecutively, in these cases the patient was classified at a lower risk category and therefore rather understaged. The amount of missing data was documented.

Patients were informed about different treatment options available and about the estimation of the treating physician, whether antibiotics were needed or not. Treatments were carried out only in agreement with the patients (informed consent). The individualized treatments were evaluated gathering information on which anthroposophic drugs and external medical applications like compresses, packs, and poultices each patient received or if the patients were treated with antibiotics and/or antipyretics. The finding process for each individual patient is based on a holistic perspective on man and earth according to the view point of anthroposophic medicine. 

For follow-up evaluation the number of leukocytes, the CRP level, the course of body temperature as well as the need for treatment on ICU, and the 30-day mortality in hospital were documented. 

For *statistical analysis t*-test for paired samples and chi-square test were applied. Missing data were replaced with the last observed value carried forward (LOCF). Calculations were performed using WinSTAT (R. Fitch Software, Germany), SAS/STAT (SAS Institute Inc., Cary, NC, USA) and SPSS (SPSS Inc., Chicago, IL, USA). A *P* value of <0.05 was considered statistically relevant. Results of the presented data were compared with data of the inpatient Pneumonia PORT Validation Cohort [32] in regard to mortality rate, the necessity to treat patients in ICU and the length of stay in hospital. statistical analysis were conducted by Thomas Ostermann, Ph.D. M.S., Professor for Research Methodology and Information Systems in Complementary Medicine, Faculty of Health, Department of Medicine, Center for Integrative Medicine, Witten/Herdecke University, Germany.

## 3. Results

Extending thirty months, 48 patients with “pneumonia” were admitted to the department of Homeotherapy in Heidenheim and treated based on anthroposophic medicine. 26 patients (19 f : 7 m) with a mean age of 65.5 years (19–90 a; SD 19.84) fulfilled the inclusion criteria “community-acquired pneumonia” (see Figure 1). The comorbidities are outlined in Table 2. 

18 of these patients showed no major comorbidities, which otherwise might mainly influence the course of the pneumonia (such as congestive heart failures, immunodeficiency), and therefore these 18 patients became the main focus for the evaluation of anthroposophic medicine (CAP-study group, see also Figure 1). The distribution of risk classification according to the pneumonia severity index (PSI) is outlined in Table 3.

On the whole 494 items could have been evaluated for calculating the PSI while 65 were missing. That counts for a missing data rate of 13.1%, from 0 to 4 data tops per patient (median 2.0). The pO_2_ and pH value were the most common missing data, followed by respiratory rate and in few cases glucose and blood urea nitrogen. 

16/18 patients were treated applying anthroposophic medicine and without the use of antibiotics; in 2/18 patients, antibiotics were applied in addition. The individualized application plan for each patient in regard to anthroposophic medication and treatment is outlined in Table 4. 

**Figure 2:**
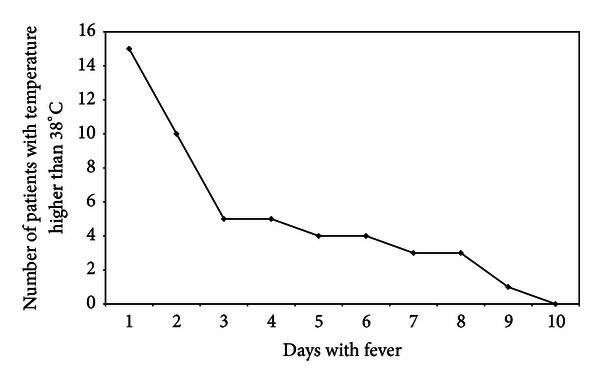
Course of temperature in group 4 (patients with AM only in the CAP-study group). Figure 2 shows the number of patients with body temperature above 38°C within the first ten days.

**Figure 3:**
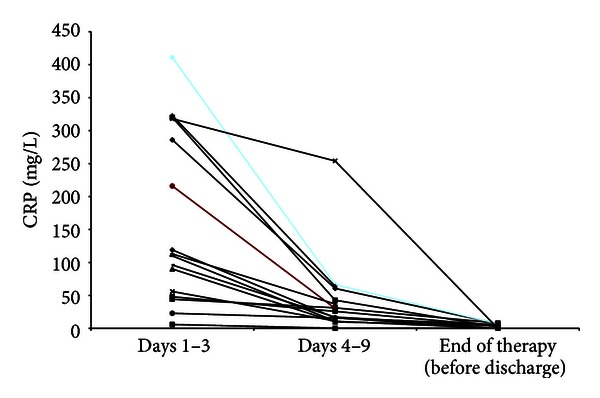
Course of CRP in group 4 (patients with AM only in the CAP-study group). CRP value 1–3 days shows the highest CRP level within the first three days, CRP value 4–9 the lowest value within this time span, and CRP before admission value at the end of treatment in hospital.

With regard to parameters which indicate efficacy of treatment (in these series *AM treatment*) the body temperature, the leukocyte blood count, and CRP levels were documented. 70% of patients were free of fever after 72 hours (3d) consecutive to the onset of AM treatment. The maximal duration of febrile body temperature amounted to 10 days (Figure 2). In one patient (who has got additionally antibiotic therapy), allopathic antipyretic therapy (Novaminsulfon acid) was applied per os over a period of 5 days. Despite two patients (out of palliative care group 3) in all patients a highly significant decrease of initially elevated CRP levels was observed (Figure 3 and Table 5) beside normalization of leukocyte blood count in cases of initial leukocytosis (Tables 8 and 9).

The mean duration in hospital within the CAP-study group (*n* = 26) was 20.2 days (Table 7). None of these patients needed to be treated on the ICU, compared to 9.2% within the control group, ranging from 4,3% to 5,9% in lower risk classes I–III, 11,4% in risk class IV, and 17,3% in risk class V whereby the duration in hospital is ranging from 5 to 11 days [32].

On the whole, one patient died for not pneumonia-related reasons (out of palliative care group 3), within the patients who fulfilled the inclusion criteria (groups 1–5, *n* = 26; 3.8%). In comparison to the control group (mortality rate of 8%), no significant difference (*P* = 0.44) within statistical analysis, using the chi-square test, was observed (Table 6). Two of the primarily excluded patients with HAP (*n* = 22, see Figure 1), who belonged to palliative care patients, died (age 90 and 91). In order to estimate whether a selection bias might influence not seeing a significant difference in comparison to the control group chi-square-test was applied also on the whole collective included (excluded patients plus groups 1–5, *n* = 48) obtaining a mortality rate of = 6.3% compared to 8.0% in the PORT control group (*P* = 0.69), indicating also no significant difference.

The CRP level was reduced significantly (*P* = 0.000) in all patients with CAP (*n* = 26, Table 5). Within the subgroup *“treated with AM only”* (group 4, CAP-study group) also a significant reduction of CRP levels was observed within 4–9 days and until discharge (*P* = 0.001 and *P* = 0.003, resp.). Within the subgroups *pretreated or additionally treated with antibiotics* (group 1 plus group 5) a significant reduction of the CRP level was only observed after 4–9 days until discharge (*P* = 0.04, Table 5).

There were no additional complications observed within the presented study.

In order to present the data most transparent, each individual course is outlined within Tables 8, 9, 10, 11, and 12 according to the groups.

## 4. Discussion

From the background of achieving high cure rates, antibiotic therapy for community-acquired bacterial pneumonia is the treatment of choice today. However with increasing resistance to antibiotics, unpleasant adverse effects and not least with rising request of patients to be treated within the scope of an integrative approach, alternative treatment options are under debate. Moreover, available data in this context is limited within the established medical literature. Therefore, the aim of the presented observational case series is to evaluate the experience in treating community-acquired pneumonia (CAP) with anthroposophic medicine (AM) within a highly specialized and well-experienced medical unit. The data of the presented observational case series are documenting the availability of an integrative treatment option for the treatment of CAP in hospital with good and comparable results in certain cases, in the context of such a specialized medical unit. Herewith, the presented study reports on unique data on a very relevant topic. However, due to the retrospective study design, the small number of patients, and a mutually not to be underestimated selection bias, the weight of conclusions for future treatment strategies in bacterial pneumonias is limited. Therefore, controlled prospective trials remain to further clarify the role of integrative medicine in the treatment of pneumonias.

Out of 48 patients with pneumonia, 26 had CAP, and 18 patients out of these were primarily treated with AM (CAP-study group, see Figure 1 and Table 3), while two of the latter got additional antibiotic treatment during their course. The individual anthroposophic treatment (as outlined in Table 4) did significantly reduce body temperature, CRP level (*P* = 0.03), and white blood cell count, while no statistical difference with regard to morbidity or mortality was observed (*P* = 0.44; *P* = 0.69), but a 2-3-fold longer hospital stay was necessary in comparison to the conventional standard antibiotic treatment of bacterial CAP in the control group [36] (Table 7). This is in line with published data concerning the antibiotic treatment of CAP [37], while there are no comparable studies on CAM or AM regarding inpatient treatment of CAP. Within the CAP study group, there was no pneumonia-related death observed, and none of the patients needed to be treated on the ICU. Anyhow, it is questionable, whether the investment of a multifold longer hospital stay—at least with regard to the costs—might be at any time convincing in order to support the integrative approach in the management of pneumonia. However, despite the economical aspect at first step, which favours the antibiotic treatment, there are also critical data on long term adverse effects in context with antibiotic and antiinflammatory treatments published [27–31, 38], such as pro-cancerous effects and/or relations to the genesis of immunological disorders, for example, in melanoma of the skin [27], in breast cancer [31], and also in hemato-oncological diseases like acute lymphatic leukaemia [30] or non-Hodgkin lymphoma [31]. The use of antibiotics and antipyretic drugs seems to play a major role in the development of allergies and/or autoimmune diseases, too [38]. But these long term sequelae of antibiotic and antipyretic/anti-inflammatory drugs as well as a potential benefit by using alternative approaches are very difficult to evaluate and therefore remain to be further investigated in future studies. From the view point of integrative medicine, the intention to mobilize human natural resources of recovery (salutogenic approach) should reduce adverse events or any other harms to the patients but still remain to be proven yet. Moreover, the rate of recurrence might be a supplemental challenging issue with regard to treatment efficacy and sustainability. Whether the character of approach (integrative and salutogenic or allopathic) may substantially influence the recurrence rate of pneumonia or other sequelae diseases should be consecutively of interest, also regarding the economic debate.

In addition, also multiresistance of pneumonia inducing bacteria has become a rising and challenging issue at present [7–11], which might be solved at least in selected patients who could be treated with anthroposophic medicine instead of antibiotics. Consecutively, selection criteria which may indicate secure application of integrative treatment options remain also to be further evaluated. In the presented course of patients with CAP the indication to additionally apply antibiotics appeared whenever a patient did not show any sign of recovering within three days after onset of treatment (like in two patients of the CAP study group) or if a progressive deterioration was obvious regarding parameters, such as dyspnea, body temperature, CRP level, or white blood cell count.

With regard to the well-validated classification of CAP into different levels of severity (PSI: pneumonia severity index), 15/18 patients of the CAP-study-group belonged to lower risk classes I–III, and all of these were treated with AM only (Table 3). Two patients of risk class IV were treated with antibiotics in addition to AM. Finally one patient classified into risk class V could also be treated with AM only. These data may show the practicability of AM in the treatment of pneumonia in principle, but neither do the low number of patients and the retrospective design allow to conclude reliable expectations on treatment results nor do they indicate certain limitations of the anthroposophic therapeutic concept. Therefore controlled prospective studies remain to be performed in order to clarify strengths and limitations of the integrative approach in the treatment of pneumonia. 

Anyhow it is worth to notice that even severe pneumonias might be approachable by applying AM only, as indicated by the patient classified in risk class V. This is in accordance with recently published data reporting a successful treatment course in a case of a 96-year-old female with severe pneumonia, lung abscess, and associated septicemia, treated with AM only (without antibiotic) [39]. Therefore, it needs years of experience as well as a time-intense dedication of the attending physicians and care team whereby external administration is mandatory in anthroposophic treatment of CAP and moreover the competence in executing the task.

Anthroposophic medicine is based on modern temporary natural science and medicine by aiming to extend these achievements with an additional holistic view on man, earth, and cosmos including the four aspects of elements and therefore intends to search for a specific individual treatment for each patient [22, 40]. AM is not intending to get in competition with modern temporary medicine but rather extending and eventually enriching it. Within a time of rising professionalised medicine with standardized clinical pathways there is almost no space for an individual treatment finding. The sketched background of AM is ordinarily excluded in conventional medicine, but within the presented case series it was intended to include all these mentioned dimensions of AM. It would be worth to further outline this characteristic process of therapy finding in an extra presentation. Further declaration of AM in detail would burst the scope of this paper and therefore remains to be outlined at other spaces.

Finally, within the context of the presented data it needs to be pointed out that integrative medicine—and as in the presented case series AM in hospital—needs a great personal effort, due to its time-intense care procedures that call for a high competence, and this might at least partly justify a prolonged hospital stay. At present, the reported data do not allow to indicate the use of anthroposophic medicine in the treatment of CAP in general. But the presented data are encouraging to further evaluate the role of integrative medicine within the treatment of CAP regarding efficacy, security, economy, and sustainability.

This case series contributed towards showing the usefulness of AM in the context with inpatient treatment of CAP. The data shows that it is possible to put selected patients with CAP on a comfortable path of recovery by treating them with AM only. Because health conscious patients in particular opt for CAM, and, in our case AM, we cannot exclude the aspects of a selection bias towards healthier patients in the presented series. Therefore, it would be particularly useful to have a larger sized controlled prospective study on the treatment of pneumonia patients with AM.

## Figures and Tables

**Table 1:** Point scoring system by Fine et al. [32] to assign the different risk classes of PSI.

Demographics	Points assigned
If male	+Age (yr)
If female	+Age (yr) − 10
Nursing home resident	+10
Comorbidity	
Neoplastic disease	+30
Liver disease	+20
Congestive heart failure	+10
Cerebrovascular disease	+10
Renal disease	+10
Physical exam findings	
Altered mental status	+20
Pulse ≥ 125/minute	+20
Respiratory rate > 30/minute	+20
Systolic blood pressure < 90mm Hg	+15
Temperature < 35°C or ≥40°C	+10
Lab and radiographic findings	
Arterial pH < 7.35	+30
Blood urea nitrogen ≥ 30 mg/dL (9 mmol/liter)	+20
Sodium < 130 mmol/liter	+20
Glucose ≥ 250 mg/dL (14 mmol/liter)	+10
Hematocrit < 30%	+10
Partial pressure of arterial O_2_ < 60 mm Hg	+10
Pleural effusion	+10

*∑* <70 = risk class II	
*∑* 71–90 = risk class III	
*∑* 91–130 = risk class IV	
*∑* >130 = risk class V	

**Table 2:** Comorbidities of all included patients in the case series (*n* = 26).

	Patients (*n*)
Heart failure	8
Cardiac arrhythmias	4
Hypertension	5
Coronary heart disease	1
Myocardial infarction	1
Aneurysm	1
Anaemia	1
Exsiccosis	1
Deep vein thrombosis	2
Pulmonary emphysema	4
Pulmonary fibrosis	2
Chronic obstructive pulmonary disease	1
Dementia	2
Psychiatric illness	2
Alcohol dependency	1
Melanoma	6
Cachexia	3
Thyroid diseases	4
Pancreatic insufficiency	1
Cirrhosis	1
Steatohepatitis	1
Others	12

**Table 3:** Patients of groups 1–5 according to risk class of PSI.

	Risk class I	Risk class II	Risk class III	Risk class IV	Risk class V	Total
No. of all patients	3	9	6	4	4	**26**
Patients treated with AM after few days antibiotics (group 1)		2		1	1	**4**
Patient with heart failure and acute decompensation (group 2)			1		1	**2**
Palliative care patients (group 3)				1	1	**2**
CAP-study group						
Patients treated with AM alone (group 4)	3	7	5		1	**16**
Patients treated with AM + antibiotics (group 5)				2		**2**

**Table 4:** Individualized application plan for each patient.

	Antibiotic	Antipyretic	Arg. m. p. D30	Echinacea D6	Ferr. sid. D20	Millefolium D4	Ferr. phos. D6	Equisetum D20	Petasites D3	Prunus spi. D3	Sticta pulm D3	Tartarus stibiatus D4	Bryonia D4	Gelomyrtol	Carb. bet. D20	Ginger	Millefol.	Cochlearia	Mustard	Potatoes
Application			s. c.	s. c./p. o.	s. c.	s. c.	s. c.	s. c.	p. o.	p. o.	p. o.	s. c.	p. o.	p. o.	s. c.	Ext.	Ext.	Ext.	Ext.	Ext.
Patient Nr																				
1			+			+			+						+		+			
2				+		+			+							+	+			
3	Pretreated		+	+				+	+	+					+	+	+			
4						+		D15	+			+	+		+	+	+		+	
5			+	+	D10	D10			+				D6				+		+	
**6**			**+**	**+**		**+**		**D3 Dil.**	**D6**			**+**			**+**	**+**	**+**			
**7**	**+**		**+**	**+**		**+**			**+**	**+**			**Dil.**		**+**	**+**	**+**			
**8**					**D10**				**D2**				**+**			**+**				
**9**	**+**	**+**				**+**														
**10**				**+**		**+**	**+**		**D6**		**+**	**+**	**+**		**+**	**+**				**+**
11	Pretreated		+	+		+			D6		+				+	+	+			
**12**				**+**		**+**			**+**	**+**	**+**	**+**		**+**	**+**				**+**	
13	Pretreated					+														
**14**			**+**	**+**		**+**			**D2 and D6**		**+**					**+**				
**15**	**Pretreated**		**+**	**+ **		**+**			**D6**	**+**			**+**			**+**				**+**
**16**			**+**	**+**		**+**			**+**	**+**	**+**		**Dil.**	**+**	**+**	**+**	**+**			
17			+			+			D2				Dil.	+	+	+	+			
**18**			**D8**	**+**		**+**			**+**	**+**			**Dil.**				**+**			
**19**					**D10**				**+**			**+**								**+**
**20**					**D10**	**+**			**+**			**+**	**+**			**+**	**+**			**+**
**21**			**+**	**+**		**+**			**+**					**+**	**+**	**+**				
**22**				**+**		**+**			**+**	**+**	**+**				**+**	**+**	**+**	**+**		
**23**			**+**	**+**		**+**	**+ **		**+**		**+**		**+**		**+**	**+**	**+**	**+**	**+**	
**24**			**+**	**+**		**+**			**+**				**+**		**+**	**+**	**+**			
**25**			**+**	**+**		**+**			**+**	**+**			**Dil.**			**+**	**+**		**+**	
**26**					**+**	**+**			**+**							**+**			**+**	

This table shows the individual therapy plan of each patient. Peroral (p.o.) and subcutaneous medication (s.c.) is outlined as well as external applications (Ext.). We omitted the illustration of convential co-medication. If the applied homeopathic potencies differed from the described in the headline, it was particularly outlined in the table. CAP-study group are bold.

**Table 5:** Statistical analysis of CRP course.

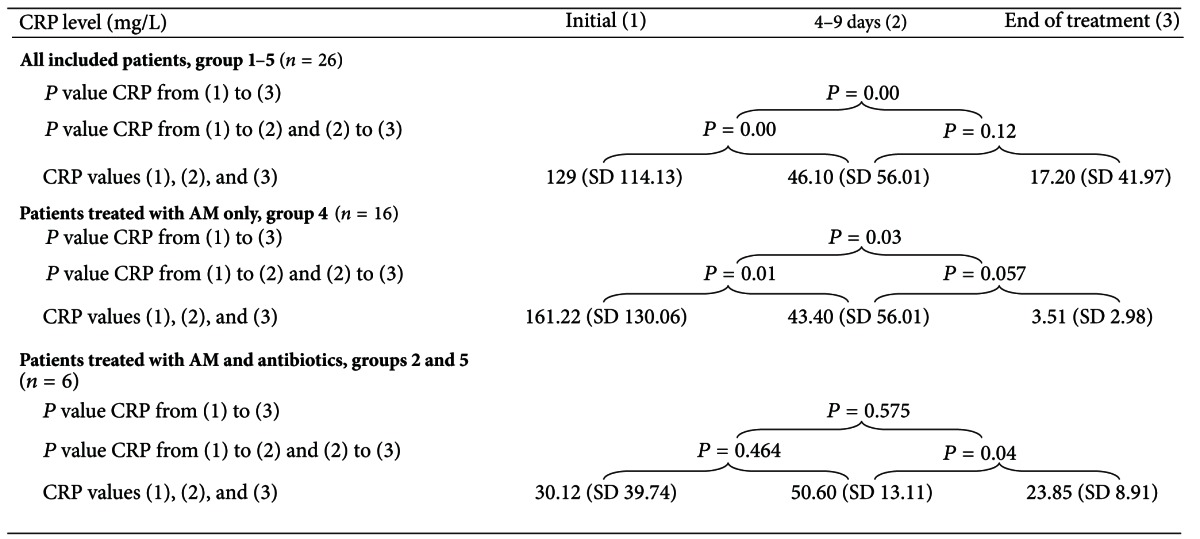

This table shows the statistical analysis of CRP decrease from the initial to the second and third value and from the second to the third value. *T*-test for paired samples was applied. (Initial, 1–3 days (1), 4–9 day (2), End of Treatment (3))

**Table 6:** Complications in comparison to control group (Pneumonia PORT Validation Cohort [32]) in regard to mortality rate and the necessity to treat patients in ICU.

	Study series	Control group (*n* = 1343)	*P* value
Treated on ICU	0/26 (0%)	124/1343 (9.20%)	*P* = 0.10
Mortality study series (*n* = 26)	1/26 (3.8%)	107/1343 (8%)	*P* = 0.44
Mortality with excluded patients (*n* = 48)	3/48 (6.25%)	107/1343 (8%)	*P* = 0.69

**Table 7:** Length of hospital stay.

	Risk class I	Risk class II	Risk class III	Risk class IV	Risk class V
Study series (*n* = 26)	19	21	9	29	23
Control group (*n* = 1343)	5	6	7	9	11

**Table 8:** CAP-study group: patients with AM treatment only (group 4).

Nr.	Sex	Age	Risk class	Temperature	First day subfebrile temp.	Leukocyte begin	Lc. end	CRP (1–3 days)	CRP (4–9 days)	CRP end	†	Comorbidities	Medical history and findings on admission	Chest X-ray
1	F	44	I	38.2	2	12.48	4.4	411	66.8	6.9		Pleurisy, hepatitis, burnout syndrome, sinusitis, and vertebral discprotrusion	For some days coughing with fever, temperature up to 40°C. Poor general condition, crackling sounds on the lungs.	Large infiltrate upper left lobe and lower right lobe.

2	F	40	I	39.9	7	Normal level		119	16	0.1		Hepatitis, sinusitis, recurringpyelonephritis, and hepatic steatosis	Sore throat and cough for 10 days, one week of fever. Poor general condition, obesity, dyspnea on exertion, chills, and crackling sounds on the lungs.	Infiltrate in the lingula of the left lung.

3	M	19	I	39.6	3	18.71	8.27	323	63.9			Pleurisy, accompanying hepatitis	Fever up to 41°C. Spastic and crackling sounds on the right side of the lung. Poor general condition.	Large infiltrate upper right lobe.

4	F	75	II	39.2	10	3.80 -	6.4	44.2	31.3	8.0		Arterial hypertension, adenoma of thethyroid	Cough and fever 3 days prior to admission. Good general condition. Crackling sounds on the lung.	Small infiltrate basolateral right.

5	F	58	II	39.2	7	Normal level		113	37			Schizophrenia, recurrent pneumonia	Cough with sputum and dyspnea 5 days prior to admission. Tachydyspnea, cyanosis of the lips, and crackling sounds on the lung.	Infiltrate lower left part of the lung.

6	M	51	II	39.7	3	2.90	4.6	96.2	17.2	3.6		Sinusitis, stomatitis, and dizziness	One week of fever up to 40°C, 2 days of strong cough with sputum. Sinusitis. Poor general condition, crackling sounds on the lungs.	Large infiltrate lower and middle lobes.

7	F	48	II	38.6	2	13.41		110	11.0	0.1		Pleurisy	Fever for one week, up to 39°C. Dry cough. Poor general condition, pleural sounds. Wheezing.	Initial: large infiltrate right middle and lower lobes.

8	F	40	II	37.0	1	Normal level		48	25.8	3.7		Depression	Cough, exhaustion, and pain in the limbs. Before admission fever, sputum, and dyspnea.	Infiltrate in the middle lobeof the lungs, bilaterally.

9	F	34	II	40.0	9	17.53	6.64	318	254	0.1		Pleurisy, burnout syndrome, and mild hyperthyreosis	One day before admission dry cough, fever up to 39°C. Poor general condition. Reduced breathing sounds.	Infiltrate lower right part of lungs.

10	M	32	II	39.9	3	17.43	4.72	320	42.8	2.1		Pleurisy, grand mal epilepsy. Recurrent pneumonia	Cough, chest pain on the right side, which got worse in the last few days, plus night sweats and a temperature up to 40.4°C. Poor general condition. Normal breathing.	Infiltrate middle lobes.

11	F	82	IIII	39.0	6	14.13	6.24	286	60.7	7.1		Chronic progressive respiratory insufficiency due to emphysema, post- tuberculosis condition with sintering of the left-sided lobe of the lungs, and arrhythmia	Poor general condition, bad nutritional state. Dyspnea.	Infiltrate left middle lobes.

12	F	67	III	38.6	2	18.47	6.64	55.6	11.4			Emphysema, chronic fibrosis of the lungs, and neurofibromatosis with cerebral microangiopathy, chronic alcoholism, and cachexia	Cough and sputum, temperature up to 39°C. At admission in a bad nutritional state, poor general condition, cyanosis, shortness of breath, neglected appearance, and crackling sounds on the lungs.	Infiltrate lower right lobe, pronounced emphysema, fibrosis, and cor pulmonale.

13	F	65	III	39.2	2	Normal level		6	0.3	0.3		Breast cancer, arterial hypertension, and arrhythmia	Fever 1d prior to admission, at admission 39.2°C, dry cough, rare sputum, weakened general condition. Crackling sounds on the lungs.	Infiltrate lower right part of lungs.

14	F	64	III	38.9	3	13.95	7.14	216	30.4			Chronic heart failure, burn-out syndrome, candidiasis, and pleurisy	One week of coughing without sputum, fever: 39-40°C, initial vomiting. Poor general condition, crackling sounds on the lungs.	Infiltrate lower right lobe.

15	F	31	III	39.4	5	Normal level		22.9	15.9	5.9		Emphysema, mental retardation, cardiac arrhythmia, mild hyperthyroidism, and mycoplasma pneumonia	One week of cough and fever, drinks little, received intravenous fluids 2 days prior to admission, poor general condition, and cachetic, crackling sounds on the lungs.	Initial: large infiltrate middle and lower lobes right and left lower lobes.

16	F	71	V	38.8	6	Normal level		90.2	9.9	4.2		Breast cancer, uterus carcinoma., primary biliary cirrhosis, and current radiotherapy	Cough, sputum. Sinusitis. Poor general condition, breathing sounds on the right side. Crackling sounds on the lungs.	Large infiltrate lower right side of the lung, pleural effusion.

Sex: F: female; M: male; risk class after Fine et al [32]. “temperature” is the highest measured temperature within the first three days outlined. First day subfebrile temperature: the first day the patient shows temperatures below 38.0°C. Leucocytes: highest number of leucocytes within the first three days. Lc. end: the count of leucocytes at discharge of the hospital. In case of normal leucocytes, no further recording performed. CRP 1st and 3rd days: highest value within the first three days as inpatients. CRP days 4 till 9: the lowest value within this time span. CRP end: CRP at end of treatment. †: Death.

**Table 9:** CAP-study group: patients with AM and additionally treated with antibiotics (group 5).

Nr.	Sex	Age	Risk class	Temperature	First day subfebrile temp.	Leukocyte begin	Lc. end	CRP (1–3 days)	CRP (4–9 days)	CRP end	†	Comorbidities	Medical history and findings on admission	Chest X-ray
1	F	79	IV	39.8	3	Normal level		94	39.0			Chronic heart failure, arterial hypertension, acute severe diarrhoea, acute hemorrhagic cystitis, decubitus ulcer (heel and coccygeal), and dehydration	Diarrhoea and fever: 39-40°C, dyspnea. Crackling sounds on the lungs, cyanotic lips. Poor general condition.	Infiltrate retrocardic left, central pulmonary congestion.

2	M	75	IV	38.9	10	Normal level		44	34.0	4.0		Acute heartattack with aneurysm of the heart during inpatient treatment pancreaticinsufficiency, condition after Billroth II resection of the stomach	38.9°C 3 days prior admission, shivering and sweating, and cough with sputum. Poor general condition. Dyspnea, crackling sound on the right side of the lungs.	Initial: no infiltrates. Control: infiltrates on the right and left sides.

Sex: F: female; M: male; risk class after Fine et al [32]. “temperature” is the highest measured temperature within the first three days outlined. First day subfebrile temperature: the first day the patient shows temperatures below 38.0°C. Leucocytes: highest number of leucocytes within the first three days. Lc. end: The count of leucocytes at discharge of the hospital. In case of normal leucocytes, no further recording was performed. CRP 1st and 3rd day: highest value within the first three days as inpatients. CRP day 4 till 9: the lowest value within this time span. CRP end: CRP at end of treatment. †: Death.

**Table 10:** Patients pretreated with antibiotics before admission (group 1).

Nr.	Sex	Age	Risk class	Temperature	First day subfebrile temp.	Leukocyte begin	Lc. end	CRP (1–3 days)	CRP (4–9 days)	CRP end	†	Comorbidities	Medical history and findings on admission	Chest X-ray
1	F	86	V	38.6	4	Normal level		99	48	29.7		Dementia, cachexia, exsiccosis, breast cancer, mildhyperthyroidism, and large pleural effusion	Recurrent fever up to 39°C while on antibiotics; multiple pretreated with antibiotics (cephalosporins, quinolone). Very poor general status, malnutrition, and attenuation of the breathing sounds.	Large pleural effusion, large infiltrate on the right lung.

2	F	57	II	37	1	Normal level		56.0	11.4	0.0		Hypothyroidism, hepatitis	Fever, cough with sputum and fatigue 3 d prior to admission. Antibiotic pretreatment of 2 d. Poor general status, cyanosis of the lips, cold sweat, and abnormal breath sounds of right lung.	Infiltrate right upper part of lungs.

3	F	68	IV	40.8	5	Normal level		25	5.0	5.2		Gastric carcinoma, hypothyroidism	One week of fever, up to 39°C 3 d prior to admission. Antibiotic pretreatment of 3 d (quinolone), no crackling sound on the lungs.	Infiltrate of the lower right segment.

4	F	66	II	38.5	8	Normal level		70	53.7	7.4		Chronic obstructive pulmonary disease(COPD), coronary heart disease, arterial hypertension, spinal syndromes with paralysis ofthe legs, and chronic heart failure (NYHA II-III)	2a of COPD with dry cough and dyspnea, temperature up to 38.5°C, and cough for one week prior to admission. Antibiotic pre-treatment of 2 d (cefaclor). Poor general status, obesity, and crackling sound on both lower parts of the lungs.	Infiltration right lower lung.

Sex: F: female; M: male; risk class after Fine et al. [32]. “temperature” is the highest measured temperature within the first three days outlined. First day sub-febrile temperature: the first day the patient shows temperatures below 38.0°C. Leucocytes: highest number of leucocytes within the first three days. Lc. end: the count of leucocytes at discharge of the hospital. In case of normal leucocytes, no further recording was performed. CRP 1st and 3rd days: highest value within the first three days as in-patients. CRP days 4 till 9: the lowest value within this time span. CRP end: CRP at end of treatment. †: Death.

**Table 11:** Patient with chronic heart failure with acute decompensation (group 2).

Nr.	Sex	Age	Risk class	Temperature	First day subfebrile temp.	Leukocyte begin	Lc end	CRP (1–3 days)	CRP (4–9 days)	CRP end	†	Comorbidities	Medical history and findings on admission	Chest X-ray
1	M	85	III	39.0	13	13.28		71	44.5	6.1		Chronic heart failure, deep vein thrombosis, and arterial hypertension	Was admitted with a deep vein thrombosis. Enlarged swollen leg. Crackling sound of the lungs. Temperature 39°C.	Infiltrate on the left side. Enlarged heart, pulmonary vascular congestion.

2	M	87	V	39.0	8	Normal level		53	16.0	<0.1		Chronic heart failure, rectal carcinoma, Pleuritis calcarea, and deep vein thrombosis	Dyspnea, fever, also thoracic pressure 3 d prior to admission. Poor general condition. Crackling sounds on the right side of the lungs.	Initial: no infiltrate, pleuritis calcarea, increased heart size, and central congestion. Control after four days: infiltrate right side infraclavicular, decrease of heart size.

Sex: F: female; M: male; risk class after Fine et al. [32]. “temperature” is the highest measured temperature within the first three days outlined. First day subfebrile temperature: the first day the patient shows temperatures below 38.0°C. Leucocytes: highest number of leucocytes within the first three days. Lc. end: the count of leucocytes at discharge of the hospital. In case of normal leucocytes, no further recording was performed. CRP 1st and 3rd days: highest value within the first three days as inpatients. CRP days 4 till 9: the lowest value within this time span. CRP end: CRP at end of treatment. †: Death.

**Table 12:** Palliative care patients (group 3).

Nr.	Sex	Age	Risk class	Temperature	First day sub-febrile temp.	Leukocyte begin	Lc end	CRP (1–3 days)	CRP (4–9 days)	CRP end	†	Comorbidities	Medical history and findings on admission	Chest X-ray
1	F	91	V	36	1	Normal level		49			†	Renal insufficiency, chronic heart failure with acute decompensation, tachyarrhythmia absoluta, and emphysema of the lungs	No fever, no cough, tachyarrhythmia absolutes (120 heart beats/minute), dyspnea, crackling sound of the lungs, and very poor general state of health (moribund).	Infiltrate lower right lobe.

2	M	90	IV	37,4	1	15.87	19.53	232.9	189	192		Chronic heart failure, acute decompensation, arrhythmia, and cachexia	Patient was already diuretically treated as outpatient for heart failure and acute decompensation. Consecutively developed an electrolyte imbalance (hypokalemia), deterioration of general status since 5 days prior to admission. 90-year-old patient with very weakened general condition and malnutrition, tachycardia (heart rate 120/min), and no increased body temperature. Ever recurring episodes of apnoea. Crackling sound on the lower right side and reduced breath sound on the right.	Large pleural infusion right lower lobe, infiltrate right lower lobe.

Sex: F: female; M: male; risk class after Fine et al. N. [32]. “temperature” is the highest measured temperature within the first three days outlined. First day sub-febrile temperature: the first day the patient shows temperatures below 38.0°C. Leucocytes: highest number of leucocytes within the first three days. Lc. end: the count of leucocytes at discharge of the hospital. In case of normal leucocytes, no further recording was performed. CRP 1st and 3rd days: highest value within the first three days as in-patients. CRP days 4 till 9: the lowest value within this time span. CRP end: CRP at end of treatment. †: Death.
